# Research Papers

**Published:** 1914-10-15

**Authors:** 


RESEARCH PAPERS. The research laboratories of Parke, Davis & Co., have just issued the second volume of the research papers for the past two years. There are twenty-two of these papers on various subjects. Such as, Cultivation of Spirocheata Pallida; Drug Influence on the Mammalian Heart; Standardization of Disinfectants, Treatment of Tetenus, etc. The volume consists of 600 pages, and is of great scientific value. It may be had of the publishers, Parke, Davis & Co., Detroit.

